# Repeat prescribing of medications: A system‐centred risk management model for primary care organisations

**DOI:** 10.1111/jep.12718

**Published:** 2017-03-31

**Authors:** Julie Price, Shu Ling Man, Stephen Bartlett, Kate Taylor, Mark Dinwoodie, Paul Bowie

**Affiliations:** ^1^ Medical Protection Society Leeds UK; ^2^ NHS Lambeth Clinical Commissioning Group London UK; ^3^ Invigilatis London UK; ^4^ Institute of Health and Wellbeing University of Glasgow UK

**Keywords:** information technology, medication safety, patient safety, primary care, risk management

## Abstract

**Rationale, aims and objectives:**

Reducing preventable harm from repeat medication prescriptions is a patient safety priority worldwide. In the United Kingdom, repeat prescriptions items issued has doubled in the last 20 years from 5.8 to 13.3 items per patient per annum. This has significant resource implications and consequences for avoidable patient harms. Consequently, we aimed to test a risk management model to identify, measure, and reduce repeat prescribing system risks in primary care.

**Methods:**

All 48 general medical practices in National Health Service (NHS) Lambeth Clinical Commissioning Group (an inner city area of south London in England) were recruited. Multiple interventions were implemented, including educational workshops, a web‐based risk monitoring system, and external reviews of repeat prescribing system risks by clinicians. Data were collected via documentation reviews and interviews and subject to basic thematic and descriptive statistical analyses.

**Results:**

Across the 48 participating general practices, 62 unique repeat prescribing risks were identified on 505 occasions (eg, practices frequently experiencing difficulty interpreting medication changes on hospital discharge summaries), equating to a mean of 8.1 risks per practice (range: 1‐33; SD = 7.13). Seven hundred sixty‐seven system improvement actions were recommended across 96 categories (eg, alerting hospitals to illegible writing and delays with discharge summaries) with a mean of 15.6 actions per practice (range: 0‐34; SD = 8.0).

**Conclusions:**

The risk management model tested uncovered important safety concerns and facilitated the development and communication of related improvement recommendations. System‐wide information on hazardous repeat prescribing and how this could be mitigated is very limited. The approach reported may have potential to close this gap and improve the reliability of general practice systems and patient safety, which should be of high interest to primary care organisations internationally.

## 1 INTRODUCTION

Preventable harm from medication‐related incidents, including those involving “repeat prescriptions,” is a significant source of concern in primary care settings worldwide.^1^, ^2^ On a daily basis in the United Kingdom, for example, over 1 million patients consult with a primary care clinician and around 2.3 million medication items are prescribed.^3^ Most National Health Service (NHS) patients prescribed pharmaceutical medications receive these as long‐term repeat prescriptions, with many taking greater than 3 medications simultaneously.^4^ Three‐quarters of patients aged 75 years and over are taking prescribed medicines, while polypharmacy is also associated with those living in higher levels of deprivation and residents in care homes.^3^, ^4^, ^5^

“Repeat prescriptions” refers to prescribed medication items that patients receive for long‐term use, normally without the need for regular or extensive monitoring or review by clinical consultation.^6^ The benefits include increased convenience for patients and reduced workloads for practices. However, it is a complex activity that requires effective communication and collaboration between clinicians, administrators, and patients to ensure that medications are appropriate, effective and prescribed at safe levels. Over the past 20 years, it is estimated that the number of repeat prescription items issued has doubled from 5.8 to 13.3 items per patient per annum.^4^ While this impacts significantly on the annual NHS budget, it also has implications for the complex management of related clinical risks, levels of avoidable patient harm, increased workloads, organisation of work in general practice, and patient experience of healthcare provision.^5^, ^6^

The patient safety implications of prescribing and medicines management in primary care are long‐established, but the scale of related “error” and “harm” is difficult to quantify depending on the definitions and interpretation of those terms.^7^ Avery and colleagues (2010) estimate a medication error rate of 7.5% across United Kingdom primary and secondary care,^8^ while Medical Protection Society (MPS) medico‐legal case data indicates that approximately 20% of litigation claims in general practice is medication‐related—the second highest reason.^9^ Previous repeat prescribing research is limited and is largely focused on types of medications,^10^ related quality improvement activity,^11^, ^12^ the views of clinicians and reception staff,^13^, ^14^ and experimental intervention studies to improve safety and efficiency.^15^ A New Zealand study by Lillis and Lord (2011) of self‐reported repeat prescribing incidents in a network of 97 general practices, and a related audit of adherence to a repeat prescribing protocol, found much variations in practice.^16^ They concluded by reaffirming that the issue is a key patient safety concern and that it will require effective practice systems, stronger patient involvement and greater pharmacy communication to reduce potential errors and harm.

Taking a systems approach to improving the quality and safety of patient care is now seen as an important prerequisite to achieving potential success.^17^ However, it is evident from the limited literature that system‐thinking using multiple educational, improvement and enabling technology interventions to monitor and improve repeat prescribing processes at frontline practice level and NHS organisational level is lacking.^18^, ^19^ In human factors science, it is accepted that errors and their consequences are the outcomes of interacting contributory factors across work systems.^20^ However, much of the aforementioned research and development on repeat prescribing has focused on preventing or minimising the risk of errors in a single part of the system only, eg, prescribing behaviours or the use of new technology. A systematic approach is necessary, therefore, to understand all safety‐critical risks and inform the design and implementation of interventions across systems to maximise opportunities to reduce harm.

Against this background, the study aimed to:

1. assess, identify and, quantifiably measure risks associated with repeat prescribing systems that can impact on patient safety and organisational performance in general medical practice;
2. provide feedback and defined actions to general practice teams on repeat prescribing system functions that can be improved and measure related impact on risk reduction;
3. provide primary care organisations with a high‐level risk monitoring mechanism for measuring repeat prescribing performance and monitoring improvements in potential harm reduction risks.

## 2 METHODS

### 2.1 Theories underlying the intervention

The proposed information technology (IT) solution tested in the study is partially informed by Deming theory of profound knowledge.^21^ This is a systems‐based management theory that suggests that organisations need to understand their business as a complex interconnected and interdependent system of elements. To consider performance and make improvements across the system, organisational leaders need access to good quality and timely data to monitor, identify, and understand variations, and facilitate continuous learning, decision‐making, and improvement.

At the practice level, the theory underpinning “audit and feedback” (defined as “*any summary of clinical performance of health care over a specified period of time aimed at providing information to health professionals to allow them to assess and adjust their performance*”)^22^ suggests that feedback on routine “repeat prescribing” performance may prompt individual clinicians and practice teams to modify and improve behaviours and systems in the area under scrutiny—particularly when compared against peers or an accepted professional standard. Performance levels (ie, the measureable reduction of risk in this study) are more likely to be improved where it is farthest away from that which is expected or desired.^22^

### 2.2 Study definitions

In the context of health and safety in the workplace and the negative consequences for people and organisations, the terms “hazard” and “risk” are closely interrelated but have slightly different theoretical meanings.^23^ However, in this study, we use both terms interchangeably to indicate identification of a potential safety problem that may impact on individual or organisational health, safety, wellbeing, or performance.

### 2.3 Setting and participants

All 48 general medical practices in NHS Lambeth Clinical Commissioning Group (an inner city area of south London in England) were recruited and participated. Practices were financially incentivised to provide time and resource to enable participation in project activity as part of the Clinical Commissioning Group (CCG) Medicines Optimisation Plan (2014/15).

### 2.4 Study design and interventions

To achieve the project aims, a multimethod study incorporating a range of educational and improvement interventions were delivered by the Medical Protection Society (MPS—Box 1) and local clinical leaders (eg, medical, pharmacy and nursing leaders):

### 2.5 “Repeat prescribing” educational workshops

Practices were invited to send 2 delegates (a General Practitioner [GP] and practice manager representative) to 1 of 3 locally‐held educational workshops conducted by an MPS clinical risk manager during October 2014. The half‐day workshops consisted of a mix of short presentations on error‐theory related to repeat prescribing [5, 6, 7, 9]**,** small group work and practical exercises. The purpose was to raise awareness of the nature and frequency of repeat medication errors and understand why care systems can fail. Delegates were also introduced to the “process mapping” method^24^ and asked to chart and risk assess their own repeat prescribing processes to identify unsafe practices, inefficiencies, the potential for standardisation and simplification, and opportunities for improvement. Practical tips on how to minimise risks and reduce preventable harm were also provided by workshop faculty.

### 2.6 Web‐based risk assessment and monitoring system

A “risk and compliance” computerised service framework (designed and customised by a commercial provider of integrated web‐based compliance solutions for a wide range of risk assessment projects) was implemented to provide oversight at individual practice and NHS organisational levels**.** The framework is engineered for the latest versions of Microsoft SQL using the. Net framework version 4, coupled with the graphics capabilities of DevExpress DXperience toolset. Expert question sets combined with relevant risk scoring methods provide organisations (eg, NHS CCGs) and clients (eg, practice teams) with real‐time risk and compliance feedback information via charts and graphs embedded in dashboards.

The framework potentially enables more efficient data collection by practice teams and automated data analysis, and the dissemination of risk compliance information than by manual methods or using multiple IT systems and people. By configuring it to meet the specific requirements of a user organisation, the framework can be deployed either in stand‐alone mode for individual assessment tasks, or in complex‐integrated services which can power corporate reporting solutions. The compliance framework is flexible and organisations can publish their own question sets with appropriate risk and compliance scoring rules. It also enables organisations to move away from existing paper‐based services while continuing with their established risk management regimes.

When deployed in this study, MPS provided each practice with a username and unique password to access its practice homepage on the web‐based system. By using the system, practices could access and act on all information collated and uploaded as part of the MPS repeat prescribing risk assessment (both previsit and postvisit) for their individual practice. The practice can address each specific risk identified during the assessment process (eg, the practice often has difficulty reading medication changes on discharge summaries leading to possibility of errors) and implementing a related improvement action (eg, formally alert local hospitals that a discharge summary is illegible or contains discrepancies), then they can update the monitoring system and view their risk reduction progress. National Health Service Lambeth CCG was also provided with a username and password to the system, which enabled the CCG to view overall results at the NHS organisational level for the 48 participating practices, as well as the results and improvement progress of individual practice teams as they addressed identified risks.

### 2.7 External repeat prescribing risk assessments

MPS Education was contracted (after an open tendering process) by NHS Lambeth Clinical Commissioning Group (CCG) Medicines Optimisation team (MOT) to undertake repeat prescribing risk assessments in all locality general practices over a 4‐month period during January to April 2015. The risk assessment involved a 2‐3 hour practice visit by 1 of MPS's clinical risk assessment facilitators (Box 2). All the risk facilitators have a clinical background and have worked or currently work in primary care, are trained and accredited by MPS in risk assessment, and are experienced in this task. The visit purpose was to undertake a comprehensive review of the practice's current systems for repeat prescribing and identify important areas of risk (as judged by MPS based on professionals' experiences and internal data) across 6 areas: prescribing processes, staff training, protocol availability and content, learning from related incidents, staffing issues, and management of drug dependent patients.

Following the visit, the MPS clinical risk assessment facilitator collated the risks and generated a report for the practice and Lambeth CCG MOT. This report included risks, improvement recommendations and a guidance section (eg, outlining the legislation of relevance to a particular risk or action). Additionally, a subjective rating system is also employed by MPS, providing a combined point score out of 400 for every identified hazard. Each hazard is risk assessed in relation to its potential impact on the following 4 domains: patient safety (100 points), clinical risk (100 points), legislation (100 points), and financial risk (100 points). An aggregated score of 150 and over denotes a high (red) risk, 100‐149 is a medium (orange) risk and 0‐99 is a low (yellow) risk. The combined points score acts as an overall measure of risk which is visibly and quantifiably reduced on the web system (at practice and CCG level) as mitigation actions are implemented.

Timescales for recommended improvement interventions for each identified risk to be implemented are colour‐coded in order of risk prioritisation: short‐term (red), medium‐term (orange), and long‐term (yellow). The report was then published on the web system within 6 weeks of the visit and linked to a “follow‐on action” section which practices could access and update as risks were addressed and improvements were implemented. When this happens a colour‐coded dashboard and pie chart illustrated a change in the risk profile and risk reduction score of the practice so that progress could be graphically visualised (ie, the system enables the practice to proactively monitor and manage their risks, track risk reduction, and also provides them with evidence of progress in improving patient safety). Similarly, at the CCG level, the same information can be viewed for individual practices or aggregated for the organisation.

### 2.8 Data collection

Data on repeat prescribing risks and identified good practices were collected on a standardised basis by the MPS clinical risk assessment facilitator during semistructured interviews with key clinical and administrative members of practice teams (guided by a tailored proforma). A review of relevant documentation was also undertaken (eg, significant event reports and practice protocols) to provide further potential insights into risks in this area. Additional data were collected during a “walk‐through‐talk‐through” verbal protocol process^25^ in the practice involving the MPS clinical risk assessment facilitator and the practice manager or practice prescribing lead (GP or practice‐based pharmacist).

### 2.9 Data analysis

Qualitative data on identified risks and actions to be taken (eg, documented short narratives) were uploaded to the aforementioned web‐based system. A basic thematic analysis^26^ of these data was undertaken by JP, who developed a coding framework and categorised and themed data on an iterative basis as analysed. PB critically reviewed the coding framework and themes generated to add validity to the findings. Disagreements were resolved by consensus. For quantitative data, basic descriptive statistics (e.g. frequency counts, means, standard deviations) were generated.

## 3 RESULTS

### 3.1 Demographics of participating practices

A total of 48 practice visits **were** undertaken during the study period. The majority of practices had specialty training accreditation (28, 58.3%) and had a list size between 5000 and 10,000 patients, while four employed a practice‐based pharmacist (8.3%) and five employed at least a single healthcare assistant (10.4%). A detailed breakdown of practice demographics is outlined in Table 1.

**Table 1:** Demographic details of participating practices (*n* = 48)

Study factor	*n*	%
Practice List Size:		
<5000	9	18.8
5001‐10000	28	58.3
10001‐15000	9	18.8
15000+	2	4.2
Number of GP Partners:		
Single‐handed	6	12.5
2‐3	27	56.2
4‐6	12	25.0
7‐9	2	4.2
10+	1	2.1
Number of Salaried GPs/Locums		
0	9	17.5
1‐2	20	34.5
3‐5	15	31.25
>5	3	6.25
Varies	1	2.1
Number of Practice Nurses		
1‐2	30	62.5
3‐4	16	33.3
>4	1	2.1
Not recorded	1	2.1
Healthcare Assistants		
Yes	5	10.4
No	43	89.6
Practice‐based Pharmacist:		
Yes	4	8.3
No	44	91.7
Specialty Training Practice Accreditation:		
Yes	28	58.3
No	20	41.7
Deprivation Payment:		
Yes	0	0.0
No	48	100.0

### 3.2 Identified good practices

Many examples of good practice in repeat prescribing were identified. Some practice teams clearly had a proactive approach to managing risks already firmly embedded in work routines, including regularly undertaking a level of risk assessment and using the methods recommended by MPS. The use of the electronic prescribing service to manage the repeat prescribing process and the implementation of a “traffic light system” to improve the clinical management of patients on medications that require regular monitoring were also noted amongst some teams. Many practices provided comprehensive information for patients on the repeat prescribing processes either in the form of patient information leaflets, online via practice websites, and on surgery notice boards. MPS clinical risk facilitators also documented strong levels of practice staff engagement, with repeat prescribing as a priority safety concern and support for the project goals.

### 3.3 Frequency of risks and risk rating scores

In total, 62 unique risks were identified across all practice repeat prescribing systems, and these were recorded on 505 occasions. This equates to a mean of 8.1 risks highlighted per practice (range: 1‐33; SD = 7.13). The mean risk rating score given to practices by MPS clinical risk assessment facilitators was 1784 points (range: 405‐3890; SD = 906.9). The individual risk score for each practice is outlined in Table 2.

**Table 2:** Aggregated risk rating scores given to individual practices after Medical Protection Society visit

Practice code	Aggregated rating score	Practice code	Aggregated rating score
**1**	2985	**25**	1210
**2**	1630	**26**	2150
**3**	1010	**27**	595
**4**	470	**28**	1460
**5**	710	**29**	2175
**6**	1570	**30**	2650
**7**	1140	**31**	1945
**8**	850	**32**	1065
**9**	3325	**33**	1210
**10**	1970	**34**	1635
**11**	1480	**35**	1005
**12**	3340	**36**	3455
**13**	2185	**37**	3345
**14**	1785	**38**	850
**15**	890	**39**	1800
**16**	3890	**40**	3495
**17**	405	**41**	685
**18**	3515	**42**	1820
**19**	1825	**43**	1430
**20**	1130	**44**	1375
**21**	1075	**45**	1505
**22**	1395	**46**	2040
**23**	2450	**47**	1465
**24**	2470	**48**	1785

### 3.4 Risk categories

A range of high risks were observed and recorded by clinical assessors that were directly related to the safe and effective operation of repeat prescribing systems (Table 3). The most hazardous high risks identified included the following: practices often having difficulty reading and interpreting medication changes on hospital discharge summaries (21, 43.8%); nonprescriber practice nurses generating prescriptions for new medications or changes to medications on their own initiative before presenting to GPs for signing (10, 20.8%); and when a repeat medication review is due, nonclinical staff being able to override the system and print a prescription (5, 10.4%).

**Table 3:** High risk “repeat prescribing” categories in descending order of severity score and frequency of occurrence in NHS Lambeth CCG general practices (*n* = 48)

High risk category	Severity score	*n*	(%)
The practice often has difficulty reading medication changes on discharge summaries leading to possibility of errors.	230	21	43.8
When a repeat medication review is due, nonclinical staff are allowed to override the system and print a subsequent prescription.	230	5	10.4
Patients on DMARDs (disease modifying antirheumatic drugs) may have prescriptions signed without assurances that they have their monitoring blood tests done.	230	4	8.3
New patient medication lists are passed to the prescription clerk who adds the medications to the computer. A prescription may be generated for a new patient without the patient having seen the doctor for a review of their medication. The additions to the computer are not checked by the doctor.	230	2	4.2
Doctors highlight medication changes or additions on letters received from hospital. Changes or additions are either done by the doctor or passed to the prescription clerk who adds or changes the medication on the computer. These entries are not checked by the doctors.	230	2	4.2
The practice has a policy of asking the prescription administrator to set up the repeat masters of new patients and to make alterations to repeat prescriptions after hospital discharge letters (rather than being reviewed by the patient's usual doctor before prescriptions are issued).	230	2	4.2
The practice does not have a recognisable system in place for the monitoring of patients taking medication that requires regular monitoring, (ie, disease‐modifying antirheumatic drugs (DMARDS), such as methotrexate and sulfasalazine and other drugs, such as amiodarone or lithium).	205	3	6.3
GPs are alerted to prescription anomalies using paper as the means of communication. There is no audit trail for the paper based communications.	200	1	2.1
Prescription pads are stored in a locked area in reception but a log of the serial numbers is not kept.	200	1	2.1
The practice nurses (nonprescribers) undertake the management of chronic diseases and may generate prescriptions for new medications or changes to medication, on their own initiation, without discussion with the GP. The prescriptions are then presented to the GPs to authorise and sign.	170	10	20.8
Medical Protection Society was informed that international normalised ratio results for patients who attend the hospital anticoagulation clinic are not sent to the practice by the clinic; the practice relies on the patient delivering their yellow book to the surgery.	170	7	14.6
There is no designated receptionist to record or generate repeat prescriptions – these are generated in the reception on an *ad hoc* basis i.e. when time permits throughout the day.	160	13	27.1
Electronic prescriptions (Electronic Prescription Service [EPS]) not being signed by the GP.	160	1	2.1
The indemnity insurance arrangements for the practice nurses is unclear.	160	1	2.1
Staff are not fully trained in the repeat prescribing process	155	12	25.0
In the case of some hospital departments and outpatient clinics, letters and discharge summaries containing medication changes are delayed by 2‐3 weeks. The patient's treatment can be delayed.	155	9	18.8
Secondary care has occasionally requested that the GP prescribe “RED list drugs,” which are not normally recommended for prescribing in primary care.	150	21	43.8

Other important risks judged “moderate” and “low,” but which may still represent a serious hazard, were also identified and categorised (Table 4). Examples included patients taking medications requiring regular monitoring medications monitored by secondary care but difficulties arising when accessing important results and information online (8, 16.7%); the repeat prescribing policy containing insufficient details of the practice system (24, 50.0%); and lack of a system to identify patients who do not request important but necessary medications (12, 25.0%). The full list of all risk categories by severity score and frequency of occurrence is outlined in Appendix 1.

**Table 4:** Selected moderate‐to‐low risk “repeat prescribing” categories in descending order of severity score and frequency of occurrence in NHS Lambeth CCG general practices (*n* = 48)

Medium risk category	Severity score	*n*	(%)
The practice does not provide patient information leaflets/cards for those patients on higher risk drugs eg, steroids and anticoagulants.	145	1	2.1
When patients are taking potentially medications requiring regular monitoring drugs, which are monitored by secondary care, there can be difficulties accessing these results and information online. This leads to increased workload obtaining the data.	140	8	16.7
There is a repeat prescribing policy, but it contains insufficient detail of the process.	130	24	50
The practice does not have a written repeat prescribing protocol.	130	8	16.7
Staff record significant events in a book or on paper, although very few are reported by non‐clinical staff.	130	5	10.4
Not all relevant staff members are involved in significant event meetings.	130	4	8.3
It is not clear whether significant events are reviewed in sufficient depth to ensure that a repeat of the event is unlikely.	130	3	6.3
There is no system in place to identify patients who do not request important medication.	125	12	25
Patients in receipt of their medication in multi‐compartment compliance aids (MCAs) are at risk when their medication is changed/altered.	125	11	22.9
The practice may not maximise the opportunities presented by patient comments on the prescribing systems.	125	1	2.1
There are occasions when the dosage instructions are “as directed”.	125	1	2.1
Accumulation of confidential paperwork left on the repeat prescribing desk. Risk of medical records not being updated and cleaners/visitors seeing confidential information.	125	1	2.1
The prescribing administrator will add and delete medication in the medical records prior to the GP reading and highlighting the discharge/out patients' letters.	125	1	2.1
Discharge summaries simply record a list of medication on discharge; there is no means of indicating medication started, medication stopped or doses changed and often no reason given for changes.	125	1	2.1
Pharmacists request the next EPS prescription for patients on 56 day supply after 5 weeks. The practice is alert to this and rejects the request.	125	1	2.1
Medication that is prescribed regularly in secondary care is not always recorded as a “hospital prescription” item on the patient's repeat prescriptions screen.	120	15	31.3
The practice faxes a significant number of prescriptions to pharmacies.	120	15	31.3
Prescriptions go missing on a daily basis.	120	8	16.7
Not all drugs prescribed by the hospital are recorded on the medication list on the clinical computer system.	120	6	12.5

**Low risk category**	**Severity score**	**(*n*)**	**(%)**
The practice has no definite system for bringing uncollected prescriptions to the attention of the prescribing doctor.	95	33	68.8
Protocols are not easily accessible for all staff.	95	3	6.3
The practice has not considered an audit of errors in prescriptions identified by local community pharmacies.	90	16	33.3
I was informed that there are pharmacies in the locality (Lambeth) who nominate patients for EPS without gaining informed consent.	90	9	18.8
Discharge summaries simply record a list of medication on discharge, there is no means of indicating medication started, medication stopped or doses changed and often no reason given for changes.	90	6	12.5
Prescriptions for controlled drugs are not signed for when collected by the pharmacy or by the patient. There is a risk that the prescription could be lost.	85	15	31.3
I was informed that no formal audit has been undertaken of warfarin prescribing.	85	9	18.8
Protocols are not retained when they have been removed from use.	60	20	41.7
A number of logistical issues with multicompartment compliance aids (MCAs) were raised, including refusal to issue unless medicines prescribed for 7 days, a specific pharmacy not receiving scripts and these needing to be reprinted.	55	17	35.4
The practice administration protocols are not signed and dated.	55	9	18.8
No audit has been undertaken of handwritten scripts issued on home visits.	50	8	16.7
The difference between an allergy and drug intolerance is not always clear in the patient record.	50	1	2.1
The practice advised that, on occasions, they have difficulty with legibility on letters from secondary care, and it is not always clear whether medication recommendations are for long or short term.	50	1	2.1
Details of the EPS are included on the website but not in the practice leaflet.	50	1	2.1
On occasions, there are issues with patients requesting NHS scripts after a private secondary care consultation.	35	15	31.3
No log of Electronic Prescription Service errors is undertaken.	35	12	25

### 3.5 System improvement recommendations

As part of the 48 practice visits, a total of 767 individual system improvement actions were recommended by MPS clinical risk assessment facilitators in 96 different categories. The mean number of recommended improvements per practice was 15.6 (SD = 8.0. range: 0‐34) with a median of 14. The top 12 main system improvement categories that are related to the highest risk category issues are outlined in Table 5. Examples include the following: formally alerting hospitals to illegible writing, anomalies and delays with discharge summaries (21, 43.8%); ensuring that monitoring requirements are added to prescription instructions (14, 29.2%); improving the current system for issuing repeat prescriptions when the review date has passed including not allowing the computer system to be overridden (5, 10.4%); and reviewing the process for generating repeat prescriptions ideally using a dedicated, trained person to complete this task in a quiet zone to aid concentration (13, 27.1%) . A full list of system improvement recommendations is outlined in Appendix 2.

**Table 5:** Top 12 actions for improvement in practices based on high risk issues identified by severity scores and frequency of occurrence (*n* = 48)

Recommendations for improvement	Severity score	*n*	%
1. Formally alert hospitals to illegible writing, discrepancies, anomalies and delays with discharge summaries and report anomalous discharge summaries to the CCG.	230	21	43.8
2. Ensure that monitoring requirements are added to prescription instructions (eg, “monthly blood test required”).	230	14	29.2
3. Review the current system of the issuing of repeats when the review date has passed. Do not allow the computer to be overridden. With the current system there is a high risk that a patient may continue to have many months of repeat medication without a review. Discuss how this could be avoided to ensure that on a subsequent request for the repeat, the GP is alerted to the number of repeats the patient has had without a medication review. You could consider entering a ‘medication review done’ code whenever the GP has reviewed the repeat prescription and reauthorised for a further 6 months (helpful for QOF).	230	5	10.4
4. Consider the process of issuing new patient prescriptions. The current procedure is risky as the patient receives a prescription signed by their new GP who has not undertaken a review of the new patient medications. The doctor is putting himself and the patient at risk by prescribing for an unknown patient, ie, drugs that another doctor has initiated/prescribed.	230	2	4.2
5. Be aware of the risks associated with repeat prescriptions that have been initiated by administrative staff. The computer audit trail will confirm the absence of any direct involvement of a clinician with the appropriate legal right to prescribe.	230	2	4.2
6. Ideally, best practice indicates that medication added to the prescription list should be done by the GP. If medication is added to the computer or changed by administration staff, it must be closely checked by the doctor afterwards; considerable care needs to be taken to ensure that all the details are correct and that it has been added to the correct patient record. The doctor has responsibility for the prescriptions he/she signs.	230	2	4.17
7. Make certain that the practice has a safe audit system to ensure that all patients taking disease‐modifying antirheumatic drugs (DMARDS), such as methotrexate and sulfasalazine and other drugs, such as amiodarone or lithium) have received the appropriate monitoring.	205	3	6.25
8. Ensure that the log of prescription serial numbers are recorded.	200	1	2.1
9. Consider communicating prescription anomalies exclusively electronically. If paper is used, ensure that there is an audit trail.	200	1	2.1
10. Review the system of the practice nurse initiating prescriptions for chronic diseases. Ensure that the system is robust. If the GPs prescribe at the recommendation of another doctor, nurse or other healthcare professional, they must satisfy themselves that the prescription is needed, appropriate for the patient and within the limits of their competence. The GPs will be responsible for any prescription they sign.	170	10	20.8
11. Discuss with the hospital warfarin clinic, how the INR results could be delivered to the practice prior to the practice issuing a prescription. Ensure that you have an anticoagulant policy in place.	170	7	14.6
12. Review the procedure of generating repeat prescriptions. This important procedure should be undertaken with due care and attention, ideally by a designated person in a quiet location where full concentration can be given to the task. Ensure that staff are fully trained and understand the importance of the repeat prescribing process.	160	13	27.1

## 4 DISCUSSION

### 4.1 Summary of main findings

The study aims were largely achieved in terms of the implementation of a package of IT and educational interventions which in combination sought to assess, identify, and measure system level risks related to repeat prescribing across a CCG. The hazard data provided can be used by practice teams to potentially drive local improvements in patient safety and monitor risk reduction progress, while providing similar aggregated and individual practice data oversight at the CCG level. Additionally, the study highlighted an important learning issue in terms of the assessment, interpretation, and prioritisation of identified risks—the high frequency occurrence of a particular risk is not necessarily an accurate indicator of its potential severity and vice versa, ie, highly significant risks may occur in low numbers across large numbers of practices.

It is clear that the approach reported is able to systematically uncover important safety issues relating to repeat prescribing, not only at practice level, but also at the interface between primary care and the other NHS care settings, which are areas of limited safety research and improvement focus.^2^, ^27^ The study data generated will also make an important contribution to knowledge of hazards related to repeat prescribing and also potentially to the design of a preliminary coding taxonomy for future classification of risks in this area. Additionally, without the external visitation and review process, it is possible, perhaps even probable, that many of the hazards highlighted would have remained undetected (ie, the latent risks described in Reason's Swiss Cheese Model^28^) until coming to light in preventable patient harm incidents or “near misses.”

Overall, the process was helpful for the CCG in identifying gaps in repeat prescribing systems across their locality and in planning strategy and financial requirements to support future developments in this area. In particular, local practices will be supported with primary to secondary care interface issues around discharge summaries and medicines reconciliation. If we assume, therefore, the reported study approach was of value in the participating CCG, at least at the practice level of identifying risks and providing feedback for improvement, then it would be fair to say that its wider implementation would have a potentially significant impact on this safety‐critical area of patient care—arguably in more effective terms than any existing improvement interventions. However, the feasibility and impact of the approach—particularly in terms of the time and resources required—are yet to be fully determined.

Next steps will involve following through the implementation of improvement recommendations with the CCG and practices and the impact of this on risk reduction in local repeat prescribing systems—to be reported in a second‐linked paper. A new technical development will be the introduction of a benchmarking comparator tool (to enhance the audit and feedback component of the intervention and strengthen the improvement), which will enable practices to monitor and compare their risk scores relative to other practices. The CCG can also monitor improvement progress and highlight those practices requiring additional support. At the GP level, the information provided by this approach will also be useful supporting evidence for medical appraisal and revalidation.

### 4.2 Strengths and limitations

There was a strong engagement from practices in the CCG area in response to incentivisation to participate, and this enabled the capture of significant levels of data on repeat prescribing risks. The MPS process can clearly identify important systems‐wide hazards and is feasible to implement at the practice level, which provides some evidence of its validity in terms of professional acceptability of the method and its potential to impact on learning and improvement. Data are collected and verified between the practice and the MPS clinical risk assessment facilitators which may strengthen data quality and the rigour of the process. Limitations include the lack of consideration to interrater calibration of the assessments and risk scoring severity undertaken by MPS clinical risk assessment facilitators, meaning actual practice risks may be under or over specified. Additionally, the study has only demonstrated the risk assessment and monitoring potential of the interventions employed but has yet to report any tangible improvements in safety systems for repeat prescribing and, therefore, measurable risk reductions at the practice and organisational level. A possible criticism of the risk management model from a resilient engineering perspective^29^ is that it is overly focused on reducing comparatively small numbers of potential safety incidents, rather than also understanding and learning from why repeat prescribing practice is safe and successful for patients in the majority of instances. Finally, the utility of the approach outlined in terms of informing successful, wider implementation beyond the innovative “early adopter” CCG organisation participating in this study is currently unknown.

### 4.3 Comparison with literature

A small number of studies report interventions to improve specific aspects of repeat prescribing systems in general practice, particularly involving targeted support of community pharmacists. For example, working in collaboration with a pharmacist and the provision of patients' annual medication data improved the quality of repeat prescribing by primary care physicians in a small study by Saastamoinen and colleagues.^30^ Wessell et al (2008) decreased “always inappropriate” and “rarely appropriate” medication prescribing in elderly patients using quarterly performance feedback reports to practices who shared a common electronic medical record system and membership of a research network.^11^ A randomised trial of clinical medication review by a pharmacist against a normal general practice review by Zermansky and colleagues (2001) demonstrated that a pharmacist can conduct effective consultations with elderly patients on reviewing their drugs.^15^ Similar research by Bond et al (2000) also found positive evidence supporting the use of pharmacists in reviewing repeat prescribing medications.^31^

Overall, intervention studies report that a combination of a medication and clinical record review by a pharmacist, together with a patient interview, may lead to reduced problems with repeat prescriptions.^8^, ^15^, ^31^, ^32^ Indeed, the UK professional bodies representing GPs and pharmacists have recently put forward joint plans recommending the benefits of having a pharmacist in practice to enhance patient safety and reduce waiting times.^33^ Pharmacists would work with GPs to resolve day‐to‐day medicines issues and, for example, assume responsibility for implementing changes to repeat prescribing systems and reducing the related workload burden of practice managers and GPs. Early indications from our study are that this is happening in the small number of participating practices who employ a pharmacist. However, while these interventions are likely to be useful as improvement strategies, there is still a major need for a systematic risk management overview of repeat prescribing on an organisational basis to monitor performance and inform learning and action over time.

### 4.4 Implications for practice and policy

The focus on this area of patient safety and the systems approach adopted will clearly have implications in terms of its contribution to demonstrating a proactive safety culture and compliance with national standards and regulators of healthcare, eg, in England, this is the Care Quality Commission (CQC). Similarly, there is arguably a greater potential to reduce the risks of formal complaints, avoidable harm incidents, medico‐legal action, and even corporate manslaughter, through participation in such a system‐wide risk management model—rather than relying on existing practice mitigation approaches, which, as the study findings demonstrate, are likely to be limited and underspecified. Additionally, at the practice level, there is limited understanding of systems thinking in resolving patient safety concerns, poor data availability and a strong medical culture of individual responsibility.^2^, ^27^

It is over a decade since a seminal policy publication recommended that system‐wide safety interventions should be a necessary part of organisational level learning and improvement in healthcare.^34^ More recently, the importance of identifying and measuring risks and harm incidents as a core patient safety obligation for organisations has been re‐stated in light of the Mid‐Staffordshire NHS Trust Public Inquiry.^35^ However, the Berwick Report has noted that there is currently limited capacity to analyse, monitor or learn from safety related information at the organisational level in all areas of healthcare.^36^ In addressing these issues, the patient safety approach described in this study offers a potentially feasible method of overcoming these barriers on a systematic basis in a particularly safety‐critical area of clinical care by providing practice and organisational data to drive improvements and hence maximise opportunities to reduce preventable harm to patients.

There is a fundamental need to recognise that implementing sustainable safety interventions in complex healthcare environments is particularly problematic.^37^ However, we would argue that our intervention has the potential to meet the key conditions that are deemed necessary to sustain a successful intervention: it should be straightforward to engage with (eg, user‐centred); it should measure and provide feedback on relevant outcomes to frontline practice (eg, quantifiable risks with linked actions for improvement are provided); and the intervention can be normalised as part of routine work to improve performance and drive cultural change.^38^, ^39^ While our study demonstrates some promise for the first 2 conditions, the third condition is predicated on the topic of repeat prescribing being judged a patient safety priority by organisational leaders, which is matched by resources to facilitate longer‐term implementation—further evaluation of the impact on learning and improvement will of course be necessary.

## 5 CONCLUSIONS

The evidence indicates that the systems‐based risk management model adopted in this study is uncovering important (and possibly previously undetected) safety concerns in the participating general practices. Currently, there is a paucity of routinely collected information available which takes a systems approach to what causes harm in this setting, why it happens and how risks can be minimised or mitigated. The combined MPS risk assessment process and the web‐based “risk and compliance” monitoring system have significant potential to prompt learning and drive safety improvements in repeat prescribing processes at the practice and NHS CCG organisational levels. The model and technologies employed should be of high interest, therefore, to UK primary care organisations prioritising the improvement of repeat prescribing‐related patient safety in their localities, as well‐being of interest internationally in similar health systems.

## FOOTNOTES

Twitter.

Follow Julie Price @juliepmps and Paul Bowie @pbnes.

## CONTRIBUTORS

Julie Price designed and project‐managed the study and contributed to the drafting and critical review of the manuscript. Shu Ling Man contributed to study design, project leadership, and the drafting and critical review of the manuscript. Stephen Bartlett designed the technical framework and computerised support system for the study and contributed to the drafting and critical review of the manuscript. Mark Dinwoodie provided leadership and expert advice to the study team and contributed to the critical review and drafting of the manuscript. Paul Bowie reviewed the literature, analysed and interpreted study data, drafted the original manuscript, collated feedback and finalised the submitted manuscript.

## CONFLICT OF INTERESTS

The authors declare no conflict of interest.

## DATA SHARING STATEMENT

No additional data are available.

## ETHICAL REVIEW

The project leadership judged this study to be a service evaluation of a quality improvement intervention, rather than research, and therefore it did not require ethical review under the “Governance Arrangements for Research Ethics Committees” in the United Kingdom. Similarly, “research” that involves NHS staff recruited as research participants by virtue of their professional roles also does not require ethical review from an established NHS research ethics committee.^40^

## APPENDIX I ALL REPEAT PRESCRIBING RISKS BY RISK CATEGORY, SEVERITY SCORE AND FREQUENCY OF OCCURRENCE.

### I.1

**Table:**

High risk	Score	N	%
The practice advised that they often have difficulty reading medication changes on discharge summaries leading to possibility of errors.	230	21	43.8
When a repeat medication review is due, non‐clinical staff are allowed to override the system and print a subsequent prescription.	230	5	10.4
Patients on DMARDs (disease modifying anti‐rheumatic drugs) may have prescriptions signed without assurances that they have their monitoring blood tests done.	230	4	8.3
New patient medication lists are passed to the prescription clerk who adds the medications to the computer. A prescription may be generated for a new patient without the patient having seen the doctor for a review of their medication. The additions to the computer are not checked by the doctor.	230	2	4.2
The practice has a policy of asking the prescription administrator to set up the repeat masters of new patients and to make alterations to repeat prescriptions after hospital discharge letters. Assurances were given that these repeat prescription master screens are always reviewed by the patient's usual doctor before prescriptions are issued.	230	2	4.2
Doctors highlight medication changes/additions on letters received from hospital. Changes or additions are either done by the doctor or passed to the prescription clerk who adds/changes the medication on the computer. These entries are not checked by the doctors.	230	2	4.2
The practice does not have a system in place for the monitoring of patients taking medications requiring regular monitoring medication.	205	3	6.3
GPs are alerted to prescription anomalies using paper as the means of communication. There is no audit trail for the paper based communications.	200	1	2.1
Prescription pads are stored in a locked area in reception; however, a log of the serial numbers is not kept.	200	1	2.1
The practice nurses (non‐prescribers) undertake the management of chronic diseases and may generate prescriptions for new medications or changes to medication, on their own initiation. The prescriptions are then presented to the GPs to authorise and sign.	170	10	20.8
MPS was informed that INR results for patients who attend the hospital anticoagulation clinic are not sent to the practice by the clinic; the practice relies on the patient delivering their yellow book to the surgery.	170	7	14.6
There is no designated receptionist to record or generate repeat prescriptions – these are generated in the reception on an ad hoc basis, ie, when time permits throughout the day.	160	13	27.1
Electronic prescriptions (Electronic Prescription Service [EPS]) not being signed by the GP.	160	1	2.1
Unsure of the indemnity arrangements for the practice nurses.	160	1	2.1
Staff are not fully trained in the repeat prescribing process.	155	12	25
In the case of some hospital departments and outpatient clinics, letters and discharge summaries containing medication changes are delayed by 2 ‐ 3 weeks. The patient's treatment can be delayed.	155	9	18.8
There have been occasions when secondary care have requested that the GP prescribe ‘RED list drugs’, which are not normally recommended for prescribing in primary care.	150	21	43.8
**Medium risk**			
The practice does not provide patient information leaflets/cards for those patients on higher risk drugs, eg, steroids and anticoagulants.	145	1	2.1
When patients are taking potentially medications requiring regular monitoring drugs, which are monitored by secondary care, there can be difficulties accessing these results and information online. This leads to increased workload obtaining the data.	140	8	16.7
There is a repeat prescribing policy but it contains insufficient detail of the process.	130	24	50
The practice does not have a written repeat prescribing protocol.	130	8	16.7
Staff record significant events in a book or on paper, although very few are reported by non‐clinical staff.	130	5	10.4
Not all staff members are involved in significant event meetings.	130	4	8.3
It is not clear whether significant events are reviewed in sufficient depth to ensure that a repeat of the event is unlikely.	130	3	6.3
There is no system in place to identify patients who do not request important medication.	125	12	25
Patients in receipt of their medication in multi‐compartment compliance aids (MCAs) are at risk when their medication is changed/altered.	125	11	22.9
Occasionally a locum doctor who has not previously worked at the practice as a registrar is employed. In this event the doctor uses a generic “locum doctor” log in; the identity of the doctor can't be readily established in the medical record.	125	1	2.1
It is not clear whether significant events are reviewed in sufficient depth to ensure that a repeat of the event is unlikely.	125	1	2.1
The practice may not maximise the opportunities presented by patient comments on the prescribing systems.	125	1	2.1
There are occasions when the dosage instructions are “as directed”.	125	1	2.1
Accumulation of confidential paperwork left on the repeat prescribing desk. Risk of medical records not being updated and cleaners/visitors seeing confidential information.	125	1	2.1
The prescribing administrator will add and delete medication in the medical records prior to the GP reading and highlighting the discharge/out patients letters.	125	1	2.1
On occasions there are issues with patients requesting NHS scripts after a private secondary care consultation.	125	1	2.1
Prescriptions issued on home visits are not entered as issued by hand on the computer record.	125	1	2.1
MPS was informed that INR results for patients who attend the hospital anticoagulation clinic are not sent to the practice by the clinic; the practice relies on the patient delivering their yellow book to the surgery.	125	1	2.1
Discharge summaries simply record a list of medication on discharge, there is no means of indicating medication started, medication stopped or doses changed and often no reason given for changes.	125	1	2.1
Pharmacists request the next EPS prescription for patients on 56 day supply after 5 weeks. The practice is alert to this and rejects the request.	125	1	2.1
Medication that is prescribed regularly in secondary care is not always recorded as a ‘hospital prescription’ item on the patient's repeat prescriptions screen.	120	15	31.3
The practice faxes a significant number of prescriptions to pharmacies.	120	15	31.3
Prescriptions go missing on a daily basis.	120	8	16.7
Not all drugs prescribed by the hospital are recorded on the medication list on the clinical computer system.	120	6	12.5
Medication updates/additions are undertaken by the medicines manager in the light of discharge summaries or outpatient follow‐up letters; these entries are checked by a GP but the checks are not documented.	120	2	4.2
GPs sign prescriptions without having access to the medical records; on occasions this also includes patients not known to them.	120	1	2.1
Repeat prescriptions can be ordered over the telephone. There is no dedicated telephone prescription line.	120	1	2.1
Staff are unsure of the process regarding the patient's choice of pharmacy for EPS.	120	1	2.1
Monitoring requirements for medications are not added to dosage instructions on repeat prescriptions.	115	14	29.2
The practice nurses are involved in review of patients with chronic diseases such as diabetes and asthma. There are no written protocols.	115	9	18.8
Significant Event Audit (SEA) meetings are held every six months for clinical staff; non‐clinical staff are not involved in the process.	115	3	6.3
In the majority of cases repeat medication is not linked to a clinical problem in the problem list.	110	14	29.2
There is no documentation of prescriptions collected by pharmacy staff.	110	11	22.9
Staff are not fully trained in learning from events.	110	9	18.8
Not all staff report incidents or near misses. Clinical staff report significant clinical incidents only.	110	8	16.7
The practice does not have any information concerning EPS scripts that are not collected from the pharmacies.	110	5	10.4
When undertaking medication reviews, patients are not routinely asked about any Over The Counter (OTC) medication they may be taking.	110	5	10.4
Prescriptions for drugs prescribed during a home visit are handwritten. These drugs are not always recorded onto the computer, resulting in an incomplete medication history for the patient.	110	2	4.2
The practice has experienced difficulty deleting prescriptions from EPS and finds that mistakes on prescriptions are difficult to rectify.	110	1	2.1
Prescriptions accidentally attached to another patient's prescription, sometimes when they have been incorrectly stapled together by the GP. This results in prescriptions being given to the wrong patient.	110	1	2.1
When repeat prescriptions are collected by a representative of the patient, there is no formal system in place to confirm that the patient has consented, the identity is not checked and the prescriptions are not signed for.	105	14	29.2
GPs are alerted to prescription anomalies using paper as the means of communication. There is no audit trail for the paper based communications.	105	14	29.2
There are occasions when the dosage instructions are “as directed”.	105	13	27.1
The difference between an allergy and drug intolerance is not always clear in the patient record.	105	9	18.8
Some messages from patients, requesting acute or additional prescription items, are passed from receptionists to doctors using paper forms. These forms are only preserved for about eight weeks.	105	8	16.7
Split prescriptions are at times considered a problem, leading to confusion for patients.	100	16	33.3
Prescription pads are stored in a locked area in reception; however, a log of the serial numbers is not kept.	100	11	22.9
Concerns were expressed regarding pharmacists delivering drugs to patients on a regular basis regardless of whether or not the patient needs the same.	100	10	20.8
The GP treats patients for drug addiction, ie, weekly methadone. He/she has not attended any specific training to undertake this role.	100	3	6.3
The practice has a free standing box for patients to place their requests for repeat prescriptions. The practice has experienced an occasion when this has been removed by a patient.	100	3	6.3
I was informed that the practice was uncertain about how to order appliances on EPS.	100	1	2.1
**Low risk**			
The practice has no definite system for bringing uncollected prescriptions to the attention of the prescribing doctor.	95	33	68.8
Protocols are not easily accessible for all staff.	95	3	6.3
The practice has not considered an audit of errors in prescriptions identified by local community pharmacies.	90	16	33.3
Pharmacies in the locality nominate patients for EPS without gaining informed consent.	90	9	18.8
Discharge summaries simply record a list of medication on discharge, there is no means of indicating medication started, medication stopped or doses changed and often no reason given for changes.	90	6	12.5
Prescriptions for controlled drugs are not signed for when collected by the pharmacy or by the patient. There is a risk that the prescription could be lost.	85	15	31.3
No formal audit has been undertaken of warfarin prescribing.	85	9	18.8
Occasionally a locum doctor who has not previously worked at the practice as a registrar is employed. In this event the doctor uses a generic “locum doctor” log in; the identity of the doctor can't be readily established in the medical record.	75	2	4.2
Protocols are not retained when they have been removed from use.	60	20	41.7
‘Scriptswitch’ is not always up to date.	60	1	2.1
A number of logistical issues with multi‐compartment compliance aids (MCAs) were raised, including refusal to issue unless medicines prescribed for seven days, a specific pharmacy not receiving scripts and these needing to be reprinted.	55	17	35.4
The practice administration protocols are not signed and dated.	55	9	18.8
The position of the signed prescriptions awaiting collection on the front desk.	55	1	2.1
No audit has been undertaken of handwritten scripts issued on home visits.	50	8	16.7
No log of EPS errors is undertaken.	50	1	2.1
Locum GPs are reluctant, and occasionally refuse, to deal with any repeat prescriptions. This can lead to delays in the process, especially when one of permanent GPs is on leave.	50	1	2.1
When repeat prescriptions are collected by a representative of the patient, there is no formal system in place to confirm that the patient has consented, the identity is not checked and the prescriptions are not signed for.	50	1	2.1
The difference between an allergy and drug intolerance is not always clear in the patient record.	50	1	2.1
The practice advised that, on occasions, they have difficulty with legibility on letters from secondary care and it is not always clear whether medication recommendations are for long or short term.	50	1	2.1
Details of the EPS are included on the website but not in the practice leaflet.	50	1	2.1
I was informed that GP2GP transfers do not allow notes greater than 5 megabytes to be transferred electronically, leading to increased time spent on gaining medical information concerning new patients.	50	1	2.1
The practice will only issue repeat prescriptions for the usual duration (28 or 56 days), for patient going on extended holidays abroad.	50	1	2.1
Pharmacists request the next EPS prescription for patients on 56 day supply after 5 weeks. The practice is alert to this and rejects the request.	45	5	10.4
On occasions there are issues with patients requesting NHS scripts after a private secondary care consultation.	35	15	31.3
No log of EPS errors is undertaken.	35	12	25
I was informed that GP2GP transfers do not allow notes greater than 5 megabytes to be transferred electronically, leading to increased time spent on gaining medical information concerning new patients.	30	2	4.2
Details of the EPS are included on the website but not in the practice leaflet.	20	19	39.6

## APPENDIX II FULL LIST OF RECOMMENDED ACTIONS FOR IMPROVEMENT BY RISK SEVERITY SCORE.

### II.1

**Table:**

Action	n	%	Risk severity score
**High risk issues**			
Formally alert hospitals to illegible writing, discrepancies, anomalies and delays with discharge summaries. Report anomalous discharge summaries to the CCG.	21	43.8	230
Review the current system of the issuing of repeats when the review date has passed. Do not allow the computer to be overridden. With the current system there is a high risk that a patient may continue to have many months of repeat medication without a review. Discuss how this could be avoided to ensure that on a subsequent request for the repeat, the GP is alerted to the number of repeats the patient has had without a medication review. You could consider entering a ‘medication review done’ code whenever the GP has reviewed the repeat prescription and re‐authorised for a further six months (helpful for QOF).	5	10.4	230
Ensure all DMARD drugs are recorded on the computer as an acute prescription so that the doctor has to review the prescription request each time and check blood monitoring is up to date. The drug could also be recorded as a repeat prescription, but not in a way that would allow it to be prescribed.	4	8.3	230
Consider the process of issuing new patient prescriptions. The current procedure is risky as the patient receives a prescription signed by their new GP who has not undertaken a review of the new patient medications. The doctor is putting himself and the patient at risk by prescribing for an unknown patient, ie, drugs that another doctor has initiated/prescribed.	2	4.2	230
Be aware of the risks associated with repeat prescriptions that have been initiated by administrative staff. The computer audit trail will confirm the absence of any direct involvement of a clinician with the appropriate legal right to prescribe. See my comments below and the section in the guidance that relates to CQC's report on prescribing after hospital discharge.	2	4.2	230
Ideally, best practice indicates that medication added to the prescription list should be done by the GP. If medication is added to the computer or changed by administration staff, it must be closely checked by the doctor afterwards; considerable care needs to be taken to ensure that all the details are correct and that it has been added to the correct patient record. The doctor has responsibility for the prescriptions he/she signs.	2	4.2	230
Ensure that staff regularly receive training on the importance of maintaining patient confidentiality.	1	2.1	220
Make certain that the practice has a safe audit system to ensure that all patients taking medications requiring regular monitoring medications have received the appropriate monitoring.	3	6.3	205
Consider communicating prescription anomalies exclusively electronically. If paper is used ensure there is an audit trail.	1	2.1	200
Ensure a log of prescription serial numbers are recorded.	1	2.1	200
Review the system of the practice nurse initiating prescriptions for chronic diseases. Ensure that the system is robust. If the GPs prescribe at the recommendation of another doctor, nurse or other healthcare professional, they must satisfy themselves that the prescription is needed, appropriate for the patient and within the limits of their competence. The GPs will be responsible for any prescription they sign.	10	20.8	170
Discuss with the hospital warfarin clinic how INR results could be delivered to the practice prior to the practice issuing a prescription. Ensure that you have an anticoagulant policy in place.	7	14.6	170
Review the procedure of generating repeat prescriptions. This important procedure should be undertaken with due care and attention, ideally by a designated person in a quiet location where full concentration can be given to the task. Ensure that staff are fully trained and understand the importance of the repeat prescribing process.	13	27.1	160
Please see guidance in the comments section regarding nurse indemnity. This will become a mandatory requirement by the NMC in 2014.	1	2.1	160
Ensure that all prescribing staff are aware that they should check at certain times of the day if there are any electronic prescriptions that require a signature. Consider putting automatic diary entries into the prescribers' calendars at first to remind them to sign scripts at certain times of the day (before and after clinics, etc).	1	2.1	160
Ensure staff involved in repeat prescribing receive appropriate training in the process.	12	25	155
Keep an incident log regarding the delay in receiving discharge summaries and consider raising the matter with the CCG, as it is likely that other practices in the area are experiencing similar problems.	9	18.8	155
Discuss with both hospital and CCG and ensure that there is clarity of whose responsibility it is to prescribe the relevant categories of drugs.	21	43.8	150
**Moderate risk issues**			
For medication where it is known that there is a higher risk, eg, steroids and anticoagulants, use supporting information for patients, such as drug information leaflets, steroid cards and tools.	1	2.1	145
Discuss the situation with Lambeth CCG and the secondary care provider to ensure ease of access to the necessary information when monitoring is undertaken in hospital but the GP has the responsibility of prescribing.	8	16.7	140
Ensure the practice's repeat prescribing protocol outlines all the good prescribing systems that take place at the practice.	24	50	130
Discuss and draw up a comprehensive repeat prescribing protocol. Ensure that all staff are trained in the procedure and have access to the protocol.	8	16.7	130
Further develop the incident reporting system, to include not only significant clinical events but all incidents and ‘near misses’. Update training for staff on the incident reporting system.	5	10.4	130
Ensure all staff receive feedback following a significant event.	4	8.3	130
Consider further training in significant events and audit, to ensure that learning has taken place to prevent an event occurring in the future. Please see the comments section.	3	6.3	130
Discuss undertaking a regular audit that would identify individuals who have failed to request their usual prescription. This might include vulnerable adults, elderly living alone, those with significant morbidity, mental health issues and others as identified by the clinicians.	12	25	125
The practice must ensure that the responsible pharmacy are notified of any medication changes affecting those patients in receipt of multi‐compartment compliance aids (MCAs). Ensure that this process is included in the practice's repeat prescribing protocol.	11	22.9	125
Ensure that locum doctors sign onto the computer system using a unique ID to enable identification of the doctor at any given time.	1	2.1	125
Consider further training in significant events and audit, to ensure that learning has taken place to prevent an event occurring in the future. Please see the comments section.	1	2.1	125
Consider using less positive comments on NHS choices, issues from PPG and complaints, specifically relating to prescribing, as opportunities to genuinely reflect on both the repeat prescribing process, patient experience of the process and if improvements to the system would be beneficial.	1	2.1	125
Avoid using “as directed” to ensure clarity for the patients and avoiding confusion with dosage instructions.	1	2.1	125
As a matter of urgency deal with the clinical correspondence on the repeat prescribing desk. After surgery hours, ensure that all prescriptions is locked away. All confidential information must be kept secure and not open to scrutiny by cleaners, unauthorised healthcare staff or the public.	1	2.1	125
As a matter of urgency review the process for adding and deleting medication in the medical records by the prescribing administrator. Ideally, best practice indicates that medication added to the prescription list should be done by the GP. If medication is added to the computer or changed by administration staff, it should be on the instruction of the GP and must be closely checked by the doctor afterwards; considerable care needs to be taken to ensure that all the details are correct and that it has been added to the correct patient record. The doctor has responsibility for the prescriptions he/she signs.	1	2.1	125
The GP should consider each request for NHS prescription, following a private consultation, on a case by case basis, using his/her clinical judgement.	1	2.1	125
Discuss with the hospital warfarin clinic how INR results could be delivered to the practice prior to the practice issuing a prescription. Ensure that you have an anticoagulant policy in place.	1	2.1	125
Record prescriptions issued on home visits as issued by hand on the patient record. Consider auditing this process in due course to check on implementation.	1	2.1	125
Through means of your LMC and Lambeth medicines optimisation team propose a review of the discharge summary template to ensure that discharge medication is comprehensibly detailed on the discharge summary.	1	2.1	125
Notify the pharmacy that the practice will not accept repeat prescription requests more than seven days before the previous prescription is due to expire except in exceptional circumstances, such as a holiday.	1	2.1	125
Ensure you have a fax policy in place and consider encouraging patients to sign up for EPS to reduce the need to fax prescriptions. MPS advises that faxing carries an increased risk of breach of confidentiality and/or faxes going astray and should be minimised.	15	31.3	120
Emphasise the importance of recording regular hospital medication on the patient's repeat screen (whilst ensuring it cannot be issued by the practice). This will ensure appropriate computer warnings of potential interactions with medication that the patient receives elsewhere.	15	31.3	120
Consider an audit of ‘missing prescriptions’ and, depending on the results, consider a system that tracks the prescription so that staff know where it should be. The practice has recently initiated electronic prescribing, a process that reduces the need for paper prescriptions and should help to reduce this problem.	8	16.7	120
Ensure that any red category or other drug prescribed by a specialist is added to the medication list with a message in the dosage instructions to indicate that it is only to be prescribed and issued by the specialist.	6	12.5	120
When a GP checks medication added or updated by the medicines manager consider making a note in the patient's record that this check has been undertaken, ie, using a Read code and free text comment.	2	4.2	120
When signing repeat prescriptions the patients' computer record should be available to enable the GP to check the accuracy of the prescription, especially those for patients unknown to the GP. The clinicians are responsible for the prescriptions that they sign.	1	2.1	120
Ensure that the system for dealing with prescription requests via the telephone is robust. Please see guidance in the comments section.	1	2.1	120
Ensure all staff are aware of the nomination process including setting, changing and cancelling a patient's nomination.	1	2.1	120
Ensure that monitoring requirements are added to prescription instructions (eg, “monthly blood test required”).	14	29.2	115
Consider developing written protocols that define the practice's approach to the management of common chronic diseases.	9	18.8	115
Expand the Significant Event Audit (SEA) meetings to include all practice staff, as necessary, and document the discussion and action plan. Ensure that action is followed up within a given time scale. Include any risk management recommendations in the appropriate protocol to ensure the same mistake is not repeated.	3	6.3	115
Diagnoses justifying continuing repeat medication, eg, hiatus hernia, should be updated on the significant active problem list and where possible linked to the relevant medication.	14	29.2	110
Consider requesting pharmacy staff to sign for the prescriptions that they collect.	11	22.9	110
Ensure staff receive training in learning from events.	9	18.8	110
Encourage staff to report all incidents and/or near misses. Consider using a ‘grumbles’ or ‘incident log’ book in reception to encourage reception staff to record near misses or incidents.	8	16.7	110
Discuss with the pharmacists and the CCG auditing uncollected scripts generated via EPS. Ensure that the pharmacies inform the GPs about uncollected medication.	5	10.4	110
Ensure that when undertaking a medication review, OTC medication is included.	5	10.4	110
Ensure that on return from home visits all handwritten prescriptions are entered as computerised prescriptions.	2	4.2	110
Review the EPS factsheet ‘Cancellation’. Whole prescriptions or individual items on a prescription can be cancelled but you cannot amend an electronic prescription once it has been signed. If something needs to be changed, the prescription or individual item must be cancelled and a new prescription generated.	1	2.1	110
When prescriptions are returned from the GP double check any that are stapled together prior to filing them ready for collection.	1	2.1	110
Consider communicating prescription anomalies exclusively electronically. If paper is used ensure there is an audit trail.	14	29.2	105
Discuss and agree a system for 3rd parties collecting prescriptions on behalf of the patients. This should include issues of consent, proof of identity and signing for the drugs, especially CDs. This should be included in the repeat prescribing policy.	14	29.2	105
Avoid using “as directed” to ensure clarity for the patients and avoiding confusion with dosage instructions.	13	27.1	105
Ensure that the patient record differentiates between an allergy and drug intolerance.	9	18.8	105
Whenever possible use the EMIS electronic messaging system to pass any messages about patients' requests for prescriptions. This approach will produce a permanent audit trail	8	16.7	105
Ensure that staff are aware that some drugs cannot be added to the EPS system and the patients are fully informed and acquainted with the system.	16	33.3	100
Ensure a log of prescription serial numbers are recorded.	11	22.9	100
To ensure that repeat medication items are not routinely delivered to patients, whether required or not, develop and agree with pharmacists a protocol to make sure only those medications required by the patient are dispatched. Excessive and over‐prescribed medications are a possible hazard to patients and a waste of resources. Please see guidance in the comments section.	10	20.8	100
Consider replacing the repeat prescribing request box with a more secure type that cannot be removed or broken into.	3	6.3	100
Ensure that the GP treating patients for drug addiction is trained, in accordance with the Department of Health's clinical guidelines for treating drug users.	3	6.3	100
Train the relevant staff on how to order appliances; discuss and confirm the method of setting the “nominated dispensing appliance contractor” in addition to the “nominated pharmacy” on EPS.	1	2.1	100
			
**Low risk issues**			
Describe in your repeat prescribing protocol a simple system for ensuring that appropriate action by the prescribing doctor is recorded when prescriptions for important medication (such as antipsychotics) are not collected.	33	68.8	95
Consider developing a central access point for all protocols such as a practice intranet or Excel spreadsheet with hyperlinks, available from the desktop of all laptops. This can be used to document the production, circulation and updating of protocols as well.	3	6.3	95
Consider an audit of errors in prescriptions identified by local pharmacies, This is a useful exercise to discuss as a team and highlight any recurrent errors that might be addressed.	16	33.3	90
If a patient has been nominated for EPS by a pharmacy without his/her consent, advise the patient that the practice can change the nomination to a pharmacy of his/her choice. Notify the pharmacy and if the problem is recurrent, notify the CCG.	9	18.8	90
Through means of your LMC and Lambeth medicines optimisation team propose a review of the discharge summary template to ensure that discharge medication is comprehensibly detailed on the discharge summary.	6	12.5	90
Consider implementing a system that requires a prescription for controlled drugs to be signed for when collected.	15	31.3	85
Consider undertaking an audit of warfarin prescribing.	9	18.8	85
Ensure that locum doctors sign onto the computer system using a unique ID to enable identification of the doctor at any given time.	2	4.2	75
Ensure out‐of‐date electronic protocols are stored in a separate folder and the date they are withdrawn recorded, rather than simply updating the electronic original.	20	41.7	60
Feedback a log of out of date advice from ‘Scriptswitch’ to the Lambeth medicines optimisation team.	1	2.1	60
Discuss with the local pharmacy issues raised about the MCAs. Keep a log of the specific issues in order to discuss with the relevant pharmacies. If issues are not resolved report the issues to Lambeth Medicines Optimisation Team. Please see guidance in the comments section.	17	35.4	55
Ensure all practice protocols are signed and dated.	9	18.8	55
Review the current positioning of the signed prescriptions awaiting collection. Discuss and agree a more secure site for these prescriptions.	1	2.1	55
Consider undertaking an audit of handwritten scripts issued on home visits to to ensure there are clear and accurate patient records.	8	16.7	50
Keep a log of and audit EPS errors, ensuring the results are shared and discussed with pharmacies, prescribing adviser and Lambeth CCG.	1	2.1	50
Review the role of the locums employed at the practice. If the practice expects the locum GP to review repeat prescriptions, this should be clearly detailed in the discussed with the locum prior to employment. If the practice requirements do require this task, then ensure that the locum is willing and competent to undertake this task, prior to employment. A copy of the repeat prescribing protocol should be provided to the locum GP prior to commencement at the practice. Access to a computer and protected time should be provided for the locum to review the prescriptions.	1	2.1	50
Discuss and agree a system for 3rd parties collecting prescriptions on behalf of the patients. This should include issues of consent, proof of identity and signing for the drugs, especially CDs. This should be included in the repeat prescribing policy.	1	2.1	50
Ensure that the patient record differentiates between an allergy and drug intolerance.	1	2.1	50
Audit the difficulties with legibility and medication difficulties and feedback the results to the Medicines Optimisation team and the hospitals.	1	2.1	50
Include details of the EPS in the practice leaflet as well as the website.	1	2.1	50
Discuss and agree an efficient method of obtaining all the records of newly registered patients.	1	2.1	50
Consider the practice policy for patients going on extended holidays abroad, ie, whether the current policy of issuing 28 or 56 days of medication may potentially put patients at risk or unnecessary expense and difficulty. Please see BMA advice Prescribing in General practice page 13. In accordance with this guidance you may wish to consider providing patients up to three months of medication when travelling abroad for an extended stay. Patients staying abroad for longer periods should be advised to seek review with the local health services (overseas) to obtain further medication. Seek clarification about local arrangements from Lambeth CCG.	1	2.1	50
Notify the pharmacy that the practice will not accept repeat prescription requests more than seven days before the previous prescription is due to expire except in exceptional circumstances, such as a holiday.	5	10.4	45
The GP should consider each request for NHS prescription, following a private consultation, on a case by case basis, using his/her clinical judgement.	15	31.3	35
Keep a log of and audit EPS errors, ensuring the results are shared and discussed with pharmacies, prescribing adviser and Lambeth CCG.	12	25	35
Discuss and agree an efficient method of obtaining all the records of newly registered patients.	2	4.2	30
Include details of the EPS in the practice leaflet as well as the website.	19	39.6	20

#### **Box 1.** About the Medical Protection Society (MPS) Education and Risk Management

MPS is the leading provider of comprehensive professional indemnity and expert advice to more than 300,000 doctors, dentists and health professionals worldwide. It is a not‐for‐profit mutual organisation, which is at the forefront of understanding risks and how to overcome them. MPS is committed to assisting members and non‐members, through education and risk management to prevent avoidable harm to patients. With a dedicated education and risk management team, MPS provide comprehensive educational programmes to healthcare professionals and teams which include the clinical risk assessments featured in this study

#### **Box 2.** Practice visit repeat prescribing issues covered by MPS clinical risk assessment facilitators during documentation reviews, ‘walk‐through/talk‐through’ and semi‐structured interviews with the manager, relevant clinicians and administrators.

- Demographic information
- Electronic Prescribing Service (EPS; where applicable)
- Staff roles and responsibilities
- Non‐clinical staff adding medication (where applicable)
- Policy for adding/altering repeat medication
- Requesting medication
- Signing prescriptions
- Prescription collection (if not EPS)
- Medication reviews
- Monitoring potentially toxic drugs
- New patients
- Formulary/Generic prescribing
- Allergies
- Dosage instructions
- Hospital prescribing
- Home visits
- Repeat Dispensing
- Security of prescriptions
- ‘Multi‐compartment Compliance Aid (MCA) dispensing’ (referred to as Medidose or Dosette boxes)
- Drug addiction
- Learning from events
  Other issues identified
